# Growth Factors for the Treatment of Ischemic Brain Injury (Growth Factor Treatment)

**DOI:** 10.3390/brainsci5020165

**Published:** 2015-04-30

**Authors:** Amara Larpthaveesarp, Donna M. Ferriero, Fernando F. Gonzalez

**Affiliations:** 1 Department of Pediatrics, University of California, San Francisco, CA 94158, USA; E-Mail: fernando.gonzalez@ucsf.edu; 2 Departments of Pediatrics and Neurology, University of California, San Francisco, CA 94158, USA; E-Mail: donna.ferriero@ucsf.edu

**Keywords:** stroke, ischemia, growth factors, erythropoietin, VEGF, IGF-1, BDNF

## Abstract

In recent years, growth factor therapy has emerged as a potential treatment for ischemic brain injury. The efficacy of therapies that either directly introduce or stimulate local production of growth factors and their receptors in damaged brain tissue has been tested in a multitude of models for different Central Nervous System (CNS) diseases. These growth factors include erythropoietin (EPO), vascular endothelial growth factor (VEGF), brain-derived neurotrophic factor (BDNF), and insulin-like growth factor (IGF-1), among others. Despite the promise shown in animal models, the particular growth factors that should be used to maximize both brain protection and repair, and the therapeutic critical period, are not well defined. We will review current pre-clinical and clinical evidence for growth factor therapies in treating different causes of brain injury, as well as issues to be addressed prior to application in humans.

## 1. Introduction

Hypoxic-ischemic injury is a significant cause of mortality and morbidity in both the adult and immature subject. While certain therapies have shown benefit in patients with stroke or hypoxic-ischemic injury that are identified early after injury, such as recombinant tissue plasminogen activator and therapeutic hypothermia, there is only partial benefit in a subset of patients with appropriate patterns and timing of injury. Brain injury progresses over a prolonged period of time through a number of different mechanisms that lead to cell damage and death. Endogenous response mechanisms following exposure to hypoxia-ischemia includes the stabilization of neuronal transcription factors hypoxia-inducible factors (HIF)-1 and 2, with increased expression of a number of downstream cytokines and growth factors. These growth factors play important roles in normal central nervous system development and function, and this increased expression following injury activates a number of signaling pathways that mediate changes in apoptosis, inflammation, angiogenesis, cell differentiation and proliferation. Despite these endogenous repair processes, significant deficits persist. Therefore, additional therapies that both lengthen the therapeutic window for treatment and enhance this long-term repair are critical for improving outcomes.

## 2. Erythropoietin

Erythropoietin (EPO) is a 30.4-kDa glycoprotein that is primarily produced in the liver in the developing fetus, and in the kidneys and liver after the neonatal period [1]. EPO plays a key role in erythropoiesis, but has also been found to have a number of other important functions in brain development and the response to injury. EPO and EPO-receptor (EPOR) are produced in the brain during gestation and during the early postnatal period, after which there is a rapid decrease to adult levels [2]. There are two categories of EPORs, a high affinity homodimer EPOR/EPOR that is present on the cell surface of erythroid precursors, and a heterodimer that pairs with other cytokine receptor molecules. The homodimeric EPOR primarily mediates the hematopoietic response, while heterodimeric EPOR appears to play an important role in mediating protective and regenerative responses, particularly in neurons and astrocytes in the brain [3].

Upon exposure to hypoxia or ischemia, HIF upregulates the production of EPO in neurons and astrocytes, with increased EPOR expression in neurons, glia, and microglia at different time points after injury [4]. Upon binding to its receptor, EPO triggers conformational changes in EPOR that activates the Janus family tyrosine protein kinase 2 (JAK-2) and MAP kinase pathways, leading to downstream activation of ERK1/2, PI3K/AKT, NFK-B, and STAT-5 [5]. Both *in vitro* and *in vivo*, EPO has been shown to regulate apoptotic and anti-apoptotic factors of the mitochondrial protein-controlled intrinsic death pathway: EPO increases the Bcl/Bax ratio in microglia and inhibits the release of apoptosis-triggering caspase-3 and -9 activation [5]. In addition to effects on cell survival, EPO promotes neurogenesis, white matter protection/regeneration, as well as anti-inflammatory and pro-angiogenic processes [6,7].

In both adult and neonatal models of ischemia, exogenous EPO significantly reduces infarct volume [8,9]. While single dose therapies improve short-term histological and behavioral outcomes, multiple dose treatment protocols result in the longest lasting beneficial outcomes [9,10,11,12,13,14]. For example, three doses of EPO given over one week, with the first dose given immediately after stroke, result in improved brain volume and cognitive function [12]. In addition, treatment initiated as late as three days after hypoxic-ischemic injury result in white matter repair and improved functional outcomes [15]. Furthermore, while treatment has resulted in improved histology and volume, it is likely that cell-type specific effects play a significant role in the beneficial response. Increased neurogenesis and oligodendrogenesis have been demonstrated in different models of ischemic brain injury [15,16,17,18,19,20]. EPO crosses the blood brain barrier (BBB) in a dose dependent manner and protects the BBB from injury-induced early VEGF-mediated leakage [21,22]. In neonatal rats, plasma and brain analyses have shown that high dose EPO, up to 5000 U/kg given intraperitoneally (IP) or subcutaneously (SC), crosses the BBB and is neuroprotective following hypoxic-ischemic injury [23]. These findings are particularly important for human clinical trials as they show that different modes of systemic administration are capable of delivery to the brain.

EPO studies in humans have shown promising results in a number of small studies, with larger randomized trials ongoing. In adults, EPO administered daily for three days immediately following stroke reduced infarct size, improved recovery of cognitive function, and decreased neurological deficits in the short-term [24]. In this study, it was noted that treatment was most effective when the first EPO dose was received within 8 h of stroke. A follow-up study in adults with stroke in the middle cerebral artery perfusion territory was less encouraging. Subjects received EPO doses at 6, 24, and 48 h after the onset of stroke symptoms, and had increased mortality (16.4%) compared to placebo (9.0%) and increased hemorrhagic complications [25]. This was attributed to the interaction of EPO with recombinant tissue plasminogen activator (rtPA), as rtPA was given later than its therapeutic window of 4.5 h [26]. In neonates, low doses of rEPO (300 or 500 U/kg) over a two-week period reduced risks for disability or death in term infants with hypoxic-ischemic encephalopathy (HIE) [27]. In a higher dose study of rEPO given over five days, neonates with mild to moderate HIE showed improved neurodevelopmental outcomes, suppressed seizures, and improved EEG results [28]. There are currently studies examining efficacy in extremely premature infants at risk for brain injury (NCT01378273) and term infants with likely hypoxic-ischemic injury (NCT01732146), as well as a study examining a long acting formulation of EPO (darbepoietin) (NCT01471015).

## 3. Vascular Endothelial Growth Factor

Vascular endothelial growth factors (VEGFs) are cytokines that stimulate angiogenesis and vasculogenesis, and are also downstream of HIF-1α activation. They include VEGF-A, VEGF-B, VEGF-C, VEGF-D and Placental-type growth factor (PlGF), with three types of VEGF receptors (VEGFR-1, VEGFR-2, VEGF-3). Most VEGF activity in the brain involves VEGF-A and VEGFR-2; however VEGF-B, PlGF, and VEGFR-1 appear to be involved to a lesser degree [29].

After an ischemic event in the brain, collateral vessel development and perfusion act as an important defense mechanism by providing arterial blood with an alternative route to the ischemic region. VEGF-A is the key mediator of arteriogenesis in the brain and is upregulated following stroke in rats, leading to increased post-ischemic angiogenesis and decreased infarct volume [30]. Following three-vessel occlusion in rats, VEGF upregulation occurred immediately and enhanced vasculogenesis for three weeks [31]. In a focal ischemic adult rat model, VEGF treatment decreased infarct volume, improved neurological deficit scores, inhibited apoptosis in the basal ganglia and cortex, increased microvessel generation, and improved the growth and proliferation of vascular endothelial cells [32].

In early stages after stroke, ischemic neurons activate astrocytes to disrupt endothelial barrier by increasing endogenous VEGF expression [33,34,35]. VEGF-A increases vascular permeability by uncoupling endothelial cell-cell junctions, resulting in BBB leakage and worse outcomes [36,37]. Thus, timing of exogenous VEGF administration is crucial for therapeutic use as studies have shown early administration of VEGF increases brain edema and infarct volume, while late application leads to decreased injury volume, increased vessel volume in the injury site, decreased glial response, and increased MBP production in adult and neonatal rodents [38,39]. In addition, VEGF-A and B have been implicated in neurogenesis following stroke, and exogenous administration has demonstrated increases in neuron number both *in vitro* and *in vivo* [40].

Human clinical studies involving the measurement of VEGF levels following stroke are ongoing. In a recently published examination of VEGF plasma values after ischemic stroke, there was a persistent increase in VEGF for three months in all subtypes of stroke [41]. There was a correlation between VEGF plasma levels and neurological/functional outcome based on ischemia subtype [41].

## 4. Brain-Derived Neurotrophic Factor

Neurotrophins are a family of growth factor proteins that are important in neuronal development and function, and have been studied as possible therapeutic options for brain injury. These include Brain-Derived Neurotrophic Factor (BDNF), Nerve Growth Factor (NGF), Glial cell line-Derived Neurotrophic Factor (GDNF), Neurotrophin-3 and Neurotrophin-4. NGF has demonstrated neuroprotection following neonatal rat hypoxia-ischemia [42], and one recent study found that intraventricular NGF may be beneficial in humans following HIE [43]. GDNF has also been shown to have neuroprotective effects following ischemic brain injury when introduced to the brain by viral vectors or GDNF-expressing cells [44]. Little research has been done using Neurotrophin-3 and Neurotrophin-4 involving ischemic brain injury, however their roles in the developing brain suggest exploration. Of the neurotrophins, BDNF has been found to be a particularly promising therapeutic candidate.

BDNF is a 13-kDa protein secreted by the postsynaptic membrane in response to neuronal excitation throughout the brain [45,46]. BDNF binds to two different cell surface receptors: a ligand-specific receptor, Tropomyosin-related kinase B, TrkB, and a general neurotrophic factor receptor, Low-affinity Nerve Growth Factor Receptor (LNGFR), also known as p75 neurotrophin receptor. When BDNF binds to TrkB, it dimerizes and autophosphorylates, leading to neuronal survival and differentiation through Ras-ERK, PI3K, and PLCγ activation and signaling [47,48]. Binding of TrkB is also associated with the regulation of long-term potentiation, plasticity, and apoptosis [47]. When BDNF binds to LNGFR, it activates a cascade of intracellular signaling pathways including NFkB, Jun kinase, and sphingomyelin hydrolysis, leading to the initiation of apoptosis [49]. Thus, temporal-specific expression of receptors is an important consideration for the exogenous administration of BDNF.

Endogenous BDNF is a key mediator of cell survival and repair in the brain after an ischemic event. Two hours following ischemia-reperfusion in adult rats, BDNF protein levels are elevated by 133%–213% in the in the cingulate and frontal cortices, and at 24 h post reperfusion, levels decrease by 40% in the striatum [50]. This rapid tapering off is thought to be due to neuronal anterograde transport of BDNF, which is hypothesized to be important for trafficking BDNF in the brain [51]. After stroke, it has also been shown that TrkB-expressing astrocytes bind and sequester vasculature-derived BDNF to promote neural precursor cell migration from the subventricular zone to the ischemic areas [52].

Numerous studies report that exogenous BDNF treatment after acute ischemic insult reduces infarct volume and significantly restores behavioral function [53,54,55]. Following subcortical ischemic stroke in adult rats, increases in oligodendrocyte differentiation and myelin formation are observed at 7 and 28 days after single-dose BDNF injected at 24 h after injury [56]. In a photothrombotic stroke model, daily intravenous injections of BDNF for five days following stroke improves sensorimotor outcomes assessed by rotorod, balance beam, and adhesive removal tests. Implantation of BDNF-transfected fibroblasts into the somatosensory cortex after stroke results in upregulation of TrkB receptors in cortical neurons of the penumbra and increased neuron survival in the cortex [57]. Intranasal administration of BDNF-overexpressing mesenchymal cells three days after stroke in neonatal rats results in reduced gray and white matter loss as well as improved motor functions at 14 days after stroke [58]. BDNF treatment has also been shown to increase neurogenesis in the dentate gyrus as well as migration of SVZ progenitor cells to the striatum of the injured hemisphere [59]. Interestingly, it has been shown that BDNF expression may be upregulated by aerobic exercise, and multiple studies have shown that exercise-induced expression is effective in enhancement of cognitive and motor function after ischemic brain injury in animal models as well as in human stroke rehabilitation studies [60,61,62,63].

Similar to VEGF, there have been no clinical trials using exogenous BDNF as a therapeutic agent, but a number of studies have examined levels following traumatic brain injury or in neurodegenerative disease. The lack of clinical trials may be secondary to the difficulty of extracting or producing appropriate amounts of BDNF for use in humans, as well as lack of high-dose studies in animal models of ischemic brain injury [64]. The Framingham study examined the correlation of VEGF and BDNF serum levels with risk of stroke [65]. Lower BDNF and higher VEGF levels were associated with increased risk of having a transient ischemic attack.

## 5. Insulin-Like Growth Factors

Insulin-like Growth Factor-1 (IGF-1) is a 7.64-kDa pleiotropic peptide that is responsible for a variety of pro-survival signaling mechanisms. While growth hormone (GH) stimulated production of IGF-1 occurs in the liver, the IGF-1 protein is found in many cell types. There are at least six different IGF-blinding proteins (IGFBPS), which are important for enhancing or blocking the effects of IGF-1, depending on the method of administration and target tissues. IGF-1 also acts through its receptor IGF-1R; however, it displays a significantly higher binding affinity for the IGFBPS, which are bound to about 99% of IGF-1 in plasma [66]. After ligand-induced autophosphorylation, IGF-1 receptor activates the PI3K-Akt pathway and the ras-raf-MEK-ERK pathway, leading to the inhibition of apoptosis and increased cell survival [67].

IGF-1 is used clinically as a treatment for growth hormone resistance related growth disorders but appears to have potential for treatment of other disorders, including ischemic brain injury. IGF-1 is widely expressed in the brain in neurons and glia, and has major roles in neurodevelopment, protection, and survival. *In vitro,* IGF-1 has been shown to inhibit glutamate, nitric oxide, and hydrogen peroxide-related apoptosis to protect both sensory and motor neurons against excitotoxicity and oxidative stress [68]. Oxygen glucose deprivation-treated microvascular endothelial cells that form the blood brain barrier also increase secretion of IGF-1 after ischemia, resulting in attenuated neuron injury [69]. Drug induced inhibition of IGF-1 and inhibition of IGF receptors on astrocytes shows that IGF-1 expression is key to astrocyte survival after H2O2-induced oxidative stress, and is key to protecting neurons from oxidative stress through astrocyte-secreted Stem Cell Factor’s interactions with IGF-1 [70].

*In vivo,* stroke increases endogenous IGF-1 expression in rats, resulting in increased proliferation of neuronal and oligodendrocyte progenitors in the SVZ and DG [71]. IGF-1 treatment decreases infarct volume compared to vehicle treated controls in rodents and sheep [72,73,74,75]. IGF-1 has also been shown to promote angiogenesis and myelination as well as neurogenesis and progenitor cell proliferation post-stroke in rats [68,71]. Intramuscular IGF-1 has been found to be an effective treatment for brain ischemia-induced muscle atrophy and further observed to increase IGF-1 expression in the CNS, decrease cortical cell apoptosis, increase activation of cortical Akt, and improve motor function scores on the parallel bar walking test [76].

In humans, IGF-1 has been studied in amyotrophic lateral sclerosis, multiple sclerosis, and Alzheimer’s disease. Studies of endogenous expression of IGF-1 after adult stroke and elderly stroke found that after ischemic injury, IGF-1 serum levels decrease significantly. All three studies found that lower levels of IGF-1 were linked to poor outcome and increased risk of death [77,78,79]. IGF-1 upregulation through exercise is also being explored in animal stroke models as well as non-ischemic human studies [80,81].

## 6. Other Growth Factors

While the previously mentioned growth factors have received the most attention as treatment for ischemic brain injury, there are many other growth factors that have been or are currently being studied. These include Progranulin (PGRN), Heparin-binding Epidermal Growth Factor-like Growth Factor (HB-EGF), Hepatocyte Growth Factor (HGF), and Granulocyte Macrophage Colony-stimulating Factor (GM-CSF). Following stroke, PGRN expression is decreased in the mouse brain, and exogenous PGRN treatment results in decreased infarct volumes and brain swelling, improved neurological scores, and reduced mortality at 24 h and seven days after stroke [82]. PGRN treatment suppresses neutrophil recruitment in the ischemic brain, leading to a reduction in NF-κB and MMP-9 activation *in vivo*, and suppresses neutrophil chemotaxis and ICAM-1 expression caused by TNF-α in endothelial cells [82]. Knockout of PGRN has been shown to cause BBB disruption and larger infarcts after stroke in adolescent mice [83]. HB-EGF administration intracerebroventricularly after focal cerebral ischemia in rats results in reduced infarct size and increased neurogenesis, while HB-EGF knockout mice have more severe injury [84,85]. HGF has been implicated in regulating angiogenesis, decreasing glial scar formation, increasing neurogenesis, promoting neuronal survival *in vivo* and *in vitro* [86]. Furthermore, GM-CSF downregulates JNK and c-jun pathway activity to decrease injury volume and decrease neuronal apoptosis following stroke in the rat [87].

## 7. Conclusions

While exogenous growth factor therapy is a promising avenue for treating ischemic brain injury, more studies are necessary to elucidate optimal timing, dosing and mode of administration, as well as possible combinations of growth factors and their effects when combined with current management strategies. Some of these therapies, like EPO, have made the leap to clinical studies and have showed promise in pilot trials, but it also necessary to consider age and sex in the translation of these therapies to the clinical setting. Because many of these growth factors have common intracellular pathway targets, exogenous administration can lead to altered expression of endogenously produced factors. For example, one hemorrhagic stroke study showed that infusion of nano-particle bound rEPO led to more than a two-fold increase in BDNF and NGF expression [88]. Further pre-clinical testing is necessary to optimize combination therapy. In addition, clarifying the mechanisms of repair will help determine therapeutic windows and other treatment strategies to maximally enhance long-term outcomes.
